# Multi‐Omic Analysis Reveals a Lipid Metabolism Gene Signature and Predicts Prognosis and Chemotherapy Response in Thyroid Carcinoma

**DOI:** 10.1002/cam4.70819

**Published:** 2025-03-22

**Authors:** Yuqin Tu, Yanchen Chen, Linlong Mo, Guiling Yan, Jingling Xie, Xinyao Ji, Shu Chen, Changchun Niu, Pu Liao

**Affiliations:** ^1^ Chongqing Medical University Chongqing China; ^2^ Department of Clinical Laboratory Chongqing General Hospital Chongqing China; ^3^ North Sichuan Medical College Nanchong China; ^4^ Department of Breast and Thyroid Surgery Chongqing General Hospital Chongqing China

**Keywords:** chemotherapy response, lipid metabolism genes, multi‐omic analysis, prognosis, thyroid carcinoma

## Abstract

**Objective:**

Lipid metabolic reprogramming is closely intertwined with the development and progression of thyroid carcinoma (TC); however, its specific mechanism remains elusive. This study aimed to investigate the association between lipid metabolism and TC progression.

**Methods:**

We employed liquid chromatography‐mass spectrometry (LC/MS) for an untargeted metabolomics analysis, comparing 12 TC patients and 12 healthy controls (HC). Additionally, we conducted the screening of differentially expressed genes (DEGs) and identified differentially expressed lipid metabolism genes (LMGs). Multi‐omic findings related to lipid metabolism were integrated to establish a prognostic risk model. The resulting risk score stratified TC patients into high‐ and low‐risk groups. Overall survival (O.S.) was assessed using Kaplan–Meier (K–M) analysis. The immune landscape was evaluated using the CIBERSORT algorithm, and chemotherapeutic response was predicted utilizing the “pRRophetic” R package.

**Results:**

Our metabolomic analysis revealed heightened lipid metabolic activity in TC, corroborated by similar findings in transcriptomic analysis. Multi‐omic analysis identified key LMGs (FABP4, PPARGC1A, AGPAT4, ALDH1A1, TGFA, and GPAT3) associated with fatty acids and glycerophospholipids metabolism. A novel risk model, incorporating these LMGs, confirmed significantly worse O.S. (*p* = 0.0045) in the high‐risk group based on TCGA_THCA. Furthermore, high‐risk TC patients exhibited lower immune cell infiltration, and predictive outcomes indicated the efficacy of potential therapeutic drugs across risk groups.

**Conclusion:**

This multi‐omic analysis underscores the potential utility of the lipid metabolism risk model in guiding clinical treatment and improving outcomes for TC patients.

## Graphical abstract

This study uncovers dysregulated lipid metabolism pathways in thyroid cancer (TC), identifying six lipid metabolism‐related genes (LMGs) as prognostic markers: FABP4, PPARGC1A, AGPAT4, ALDH1A1, GPAT3, and TGFA. A prognostic risk model based on these LMGs demonstrates superior predictive performance for overall survival. Dysregulated pathways, including the TCA cycle and glycerophospholipid metabolism, are implicated in TC progression. The risk model correlates with immune cell infiltration and chemotherapy sensitivity, guiding personalized therapeutic strategies. These findings highlight the potential of targeting dysregulated lipid metabolism pathways and LMGs for novel therapeutic interventions in TC.

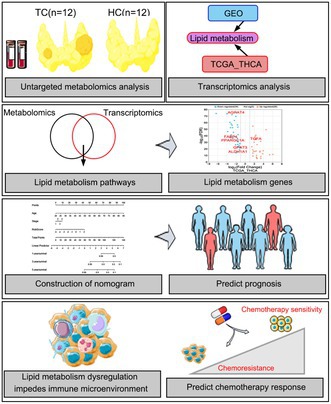

## 1 Introduction

Thyroid carcinoma (TC) stands as the most prevalent malignancy within the endocrine system. TC encompasses various histological types, including differentiated thyroid carcinoma (DTC) such as papillary, follicular, and Hürthle cell carcinomas, as well as medullary and anaplastic thyroid carcinomas (MTC and ATC). Among these, papillary thyroid carcinoma (PTC) represents its predominant subtype, accounting for approximately 84% [1, 2, 3]. According to 2024 cancer statistics, TC constituted 3% of new cancer cases in women [4]. The incidence of TC has experienced a significant surge in China since 2000 [5]. Notably, approximately 2%–3% of TC cases do not respond to standard treatment, and patients with advanced tumors exhibit lower survival rates [6, 7, 8]. Currently, widely adopted clinicopathological risk factors lack efficacy in discerning the degree of disease risk in TC patients. Given the advent of omics technologies such as transcriptomics and metabolomics, the application of multi‐omics analysis in TC has emerged as a reliable approach to systematically search for prognostic signatures and predict targeted therapy.

At present, surgery stands out as the most effective treatment for TC; however, the challenge of overdiagnosis remains significant. Biomarkers such as BRAF^V600E^, RAS, and TP53 mutations have been recommended by The American Thyroid Association (ATA) and serve as adjuncts for diagnosing, treating, and predicting prognosis in TC [9, 10]. However, when the cytological examination of fine‐needle aspiration (FNA) samples is indeterminate, the positive predictive value of the pattern recognition approaches of molecular classifiers is 42% to 68% [11, 12]. Despite their utility, ultrasound and CT diagnoses have limitations, presenting a clinical challenge for preoperative diagnosis [13]. Studies have demonstrated that different risk groups can be distinguished by detecting transient changes in metabolites [14, 15]. The intricate relationship between metabolism reprogramming and tumor progression is evident, with lipid accumulation being linked to the promotion of tumorigenesis, proliferation, and distant metastasis. Cancer cells exploit lipid metabolism to modulate the function of stromal and immune cells, providing a strategic advantage in evading therapy and promoting relapse [16, 17]. A recent study has highlighted the promotion of malignant proliferation in TC through enhanced lipid metabolism [18]. In addition, multi‐omics data analysis has unveiled that augmented fatty acid metabolism furnishes supplementary energy and substrates vital for the progression of TC [19]. However, the tumorigenic effects and molecular mechanisms underlying lipid metabolism in TC remain undetermined.

Here, we utilized transcriptomic and untargeted metabolomic data to analyze co‐enrichment pathways and genes related to lipid metabolism in DTC. We assessed the prognostic potential of six identified lipid metabolism genes (LMGs) as biomarkers. Subsequently, a nomogram was constructed, and its performance was validated. Furthermore, we elucidated the association between the risk model and tumor‐infiltrating immune cells (TIICs) and predicted the sensitivity of potential therapeutic drugs in the TCGA_THCA database. Finally, we validated the expression of these identified genes in various TC cell lines. To sum up, our investigation uncovered dysregulated lipid metabolism pathways and LMGs in TC, offering novel insights for clinical prognosis assessment and the development of personalized metabolic therapy strategies.

## 2 Materials and Methods

### 2.1 Setting and Overview

The incidence of TC, particularly DTC, is steadily increasing. Despite advances in diagnosis and treatment, the complexities of TC progression remain poorly understood. This study elucidates the role of lipid metabolism in TC by integrating transcriptomic and metabolomic data, identifying key LMGs, and developing a novel prognostic risk model. We examined the correlation between the risk model and immune cell infiltration and predicted the efficacy of potential therapeutic drugs in the TCGA_THCA database.

### 2.2 Data Collection

Four microarray datasets, namely GSE3678, GSE33630, GSE29265, and GSE3467, were retrieved from the GEO database (https://www.ncbi.nlm.nih.gov/geo/). Additionally, RNA‐Seq data and clinical information for TCGA_THCA were obtained from the TCGA database (https://portal.gdc.cancer.gov/). A detailed description of each dataset is provided in Table S1 for reference. The raw data underwent rectification and normalization using the “limma” package in R software (x64 4.2.0). For a visual representation of the workflow, refer to Figure S1.

### 2.3 Serum Sample Preparation and Metabolomics Profiling

A total of 24 subjects were enrolled for LC/MS analysis, including 12 TC patients and 12 healthy controls (HC). Serum samples were collected from both groups. Clinical data, including age, gender, TNM stage, and so on, were documented (Table S2). TC diagnosis was confirmed through histopathological examination post‐surgery. The analysis was performed utilizing an ultra high performance liquid chromatography (UHPLC) system (Vanquish UHPLC, Thermo) coupled with an Orbitrap mass spectrometer (Q Exactive HF) at Shanghai Applied Protein Technology Co. Ltd. Both electrospray ionization (ESI) positive and negative modes were utilized to ensure comprehensive coverage of the metabolome. HILIC separation was performed using an ACQUITY UPLC BEH Amide column (2.1 mm × 100 mm, 1.7 μm; Waters, Ireland). The raw MS data underwent preprocessing using ProteoWizard MSConvert before being imported into the XCMS software. Quality control (QC) samples, generated from combined aliquots of each processed sample, were injected every 10 samples to monitor data acquisition consistency and enable subsequent abundance normalization [20]. We utilized CAMERA (Collection of Algorithms of MEtabolite pRofile Annotation) for annotating isotopes and adducts. Only variables with more than 50% of nonzero measurement values in at least one group were retained in the extracted ion features. Metabolite compound identification relied on comparing accurate m/z values (< 10 ppm) and MS/MS spectra with an in‐house database established using available authentic standards [21]. This study received approval from the Ethics Committee of Chongqing General Hospital, and all participating patients did so voluntarily.

### 2.4 Metabolomics Data Analysis

Following sum‐normalization, the processed data underwent analysis using the R package (ropls), including Pareto‐scaled principal component analysis (PCA) and orthogonal partial least squares discriminant analysis (OPLS‐DA). Both PCA and OPLS‐DA models were employed to investigate differentially abundant metabolites (DMs) between TC and HC. In the OPLS‐DA model, the variable importance in the projection (VIP) value for each variable was calculated to indicate its contribution to classification. Further selection of DMs relied on the *p* value or fold change obtained from univariate analysis. After abundance matrix correction, Student's *t*‐test was used to determine the significance of differences between two independent sample groups. To identify significant changes, a threshold of VIP > 1 and a *p* value < 0.05 were applied. Enrichment analysis of DMs was carried out using the OECloud tools available at https://cloud.oebiotech.com, with the cut‐off criteria set at a *p* value < 0.05.

### 2.5 Bioinformatic Analysis

The identification of DEGs utilized the R package “limma” (version 3.50.3) [22], with statistically significant values defined by a threshold of |Log2FC| > 1 and adjusted *p* < 0.05. The VennDiagram package (version 1.7.3) was employed to analyze the overlap among the four GEO datasets. Result diagrams, such as volcano plots, heatmaps, and forest plots, were generated using the online platform for data analysis and visualization at https://www.bioinformatics.com.cn [23]. Functional enrichment analysis, focusing on genes related to lipid metabolism pathways, was conducted using the Sangerbox platform to access Gene Ontology (GO) and Kyoto Encyclopedia of Genes and Genomes (KEGG) databases [24]. A significance threshold of *p* < 0.05 and false discovery rate (FDR) < 0.25 was applied. Prognostic analyses were conducted using the R packages “survival” (version 3.4.0) and “survminer” (version 0.4.9) for both univariate and multivariate assessments of independent prognostic values of specific gene signatures. A least absolute shrinkage and selection operator (LASSO) regression analysis was conducted using the R package “glmnet” (version 4.1–7). Subsequently, a Cox proportional hazards regression prognostic model was constructed by incorporating predictive prognostic genes into the regression equation to obtain risk scores, expressed as: risk score = SUM [gene expression × coefficient]. Risk assessment was conducted using the R packages “ggrisk” (version 1.3) and “rms” (version 6.3–0). Samples in the TCGA_THCA cohort were categorized into high‐ or low‐risk groups according to their median risk scores. Receiver operating characteristic (ROC) and Kaplan–Meier (K–M) analyses were performed between the two groups. The R package “survival” was employed to compare overall survival (O.S.). The prognostic performance of the model was evaluated at 1‐, 3‐, and 5‐year using the “pROC” package to calculate the ROC and area under the curve (AUC).

### 2.6 Development of Nomogram and Gene Set Enrichment Analysis (GSEA)

Age, stage, and risk score were utilized to construct a nomogram employing R packages such as “survival”, “rms” (version 6.3–0), “nomogramFormula,” and “ggplot2.” Calibration curves for the nomogram were generated to evaluate the concordance between predicted and actual survival. To investigate the enrichment of signaling pathways and biological processes related to LMGs between high‐ and low‐risk groups, GSEA was employed (http://software.broadinstitute.org/gsea/index.jsp). The algorithm's parameters included a minimum gene set size of 5 and a maximum gene set size of 5000.

### 2.7 Immune Infiltration Analysis and Gene Set Cancer Analysis (GSCA)

The proportion of 22 immune cell types in each THCA sample from the TCGA_THCA database was computed using CIBERSORT [25], a deconvolution algorithm. To ensure result reliability, 1000 permutations were performed, and samples with *p* < 0.05 were retained. Estimation of TIICs and lipid metabolism biomarkers was based on the risk signature. The Wilcoxon rank‐sum test was used to examine the difference in immune cell proportions between high‐ and low‐risk groups.

Correlation analysis between LMGs and immune cells was conducted using the “psych” R package (version 2.3.9) to visualize their relationship. Additionally, the GSCA platform was employed for immunogenomic gene set cancer analysis [26], focusing on the correlation between variations in LMGs and the abundance of immune cells.

### 2.8 Prediction the Sensitivity of Potential Therapeutic Drugs Based on Risk Stratification

The half‐maximal inhibitory concentration (IC_50_) of each THCA patient from the TCGA_THCA database was predicted utilizing the “pRRophetic” R package, leveraging data from the Genomics of Drug Sensitivity in Cancer (GDSC) dataset. This approach was employed to evaluate therapeutic drug sensitivity [27]. With the emerging concept of personalized treatment for TC, predicting treatment response is crucial. Chemotherapy response for 251 drugs was forecasted across different risk groups. While most are not FDA‐approved for certain types of TC, they are recommended by the National Comprehensive Cancer Network (NCCN) [28]. We selected 12 drugs relevant to TC treatment, including sorafenib [29], dabrafenib [30], trametinib [31], Lenvatinib [32], axitinib [33], doxorubicin [34], gemcitabine [35], imatinib [36], paclitaxel [37], pazopanib [38], sunitinib [39], and bexarotene [40].

### 2.9 Cell Lines and Cell Culture

The human thyroid follicular epithelial cell line (Nthy‐ori3‐1) was obtained from iCellbioscience (Shanghai, China), and the human TC cell lines (BCPAP, KTC‐1, and TPC‐1) were purchased from Procell (Wuhan, China). All cells were cultured in RPMI‐1640 (Gibco) supplemented with 10% fetal bovine serum (FBS) and 1% penicillin/streptomycin (P/S), and maintained in a humidified incubator with 5% CO_2_ at 37°C. Authentication of all cell lines was performed using a Short Tandem Repeat (STR) assay.

### 2.10 Real‐Time Quantitative PCR (RT‐qPCR) and Western Blot

Total RNA from cell samples was extracted using the RNAiso Plus reagent (#9108, TaKaRa), and cDNA was synthesized using the Prime Script RT Reagent Kit (#RR047A, TaKaRa). For RT‐qPCR analysis of selected gene expression, the TB Green Premix Ex Taq II (#RR820A, TaRaKa) was employed, with GAPDH serving as the endogenous control. The QuantStudio5 real‐time PCR system (Applied Biosystems) was used to measure relative mRNA expression levels, with data analysis performed using the 2^(−ΔΔC(T))^ method. Each sample was examined in triplicate, and the experiment was repeated independently three times. Primer sequences are provided in Table S3.

Cell proteins were extracted using RIPA lysis buffer (#P0013B, Beyotime), and the protein concentration was determined using a BCA protein assay kit (#WB6501, NCM Biotech). Equal amounts of proteins were isolated using SDS‐PAGE and blotted onto a polyvinylidene fluoride membrane (Millipore, USA), followed by blocking with 5% nonfat milk for 1.5 h at room temperature. Membranes were then incubated overnight with the designated primary antibodies at 4°C, followed by incubation with the secondary antibody for 1 h. Signal detection was performed using the enhanced chemiluminescence (ECL) detection kit (#P2200, NCM Biotech) with an automatic chemiluminescence/fluorescence image analysis system (Bio‐Rad, USA). The antibodies used are listed in Table S4.

### 2.11 Statistical Analysis

Statistical analyses were conducted using R 4.2.0 (University of Auckland, New Zealand), SPSS 25.0 (IBM, USA), and GraphPad Prism 8.0 (San Diego, CA, USA). The data were presented as the mean ± standard error of the mean. Significant differences between the means of two groups were determined using Student's *t*‐test. Spearman's correlation coefficient was employed for correlation analysis, and the Wilcoxon test was used to compare data from different groups. All analyses were two‐tailed, and the *p* value < 0.05 was considered indicative of statistically significant results.

## 3 Results

### 3.1 Screening and Analysis of 62 Differential LMGs

**FIGURE 1:**
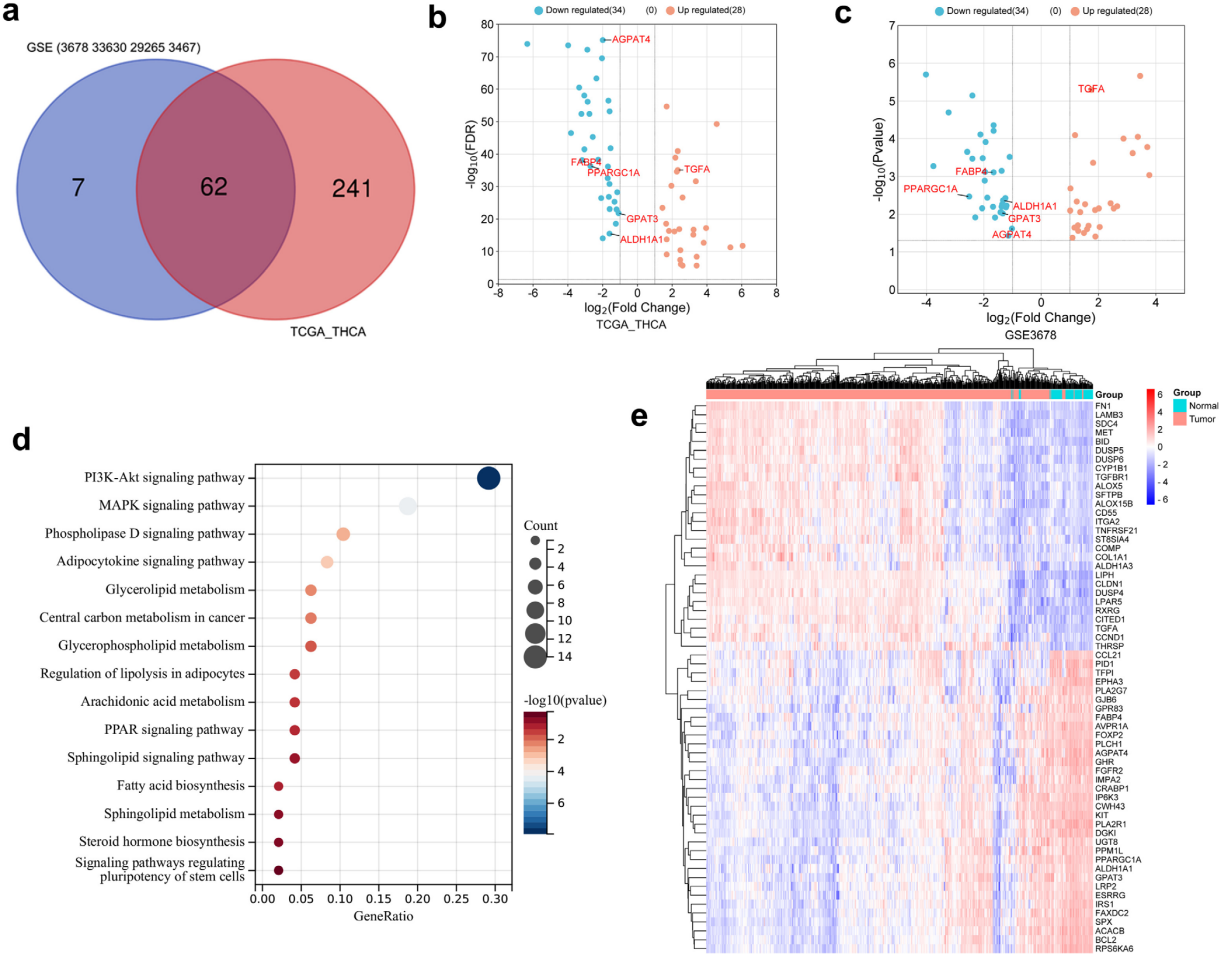
Differential expression analysis of lipid metabolism genes (LMGs) and functional annotation in thyroid carcinoma (TC). (a) Venn diagram of intersection differential LMGs between GSE3678, GSE33630, GSE29265, GSE3467 and TCGA_THCA datasets. (b, c) Volcano plot representing differential expression LMGs in the TCGA_THCA/GSE3678 datasets. The red dots in the plot represent upregulated genes, blue dots represent downregulated genes with statistical significance. (d) KEGG enrichment analysis of the differentially expressed LMGs. (e) Heatmap visualizing the expression of LMGs between TC and healthy control (HC) in TCGA_THCA dataset.

**FIGURE 2:**
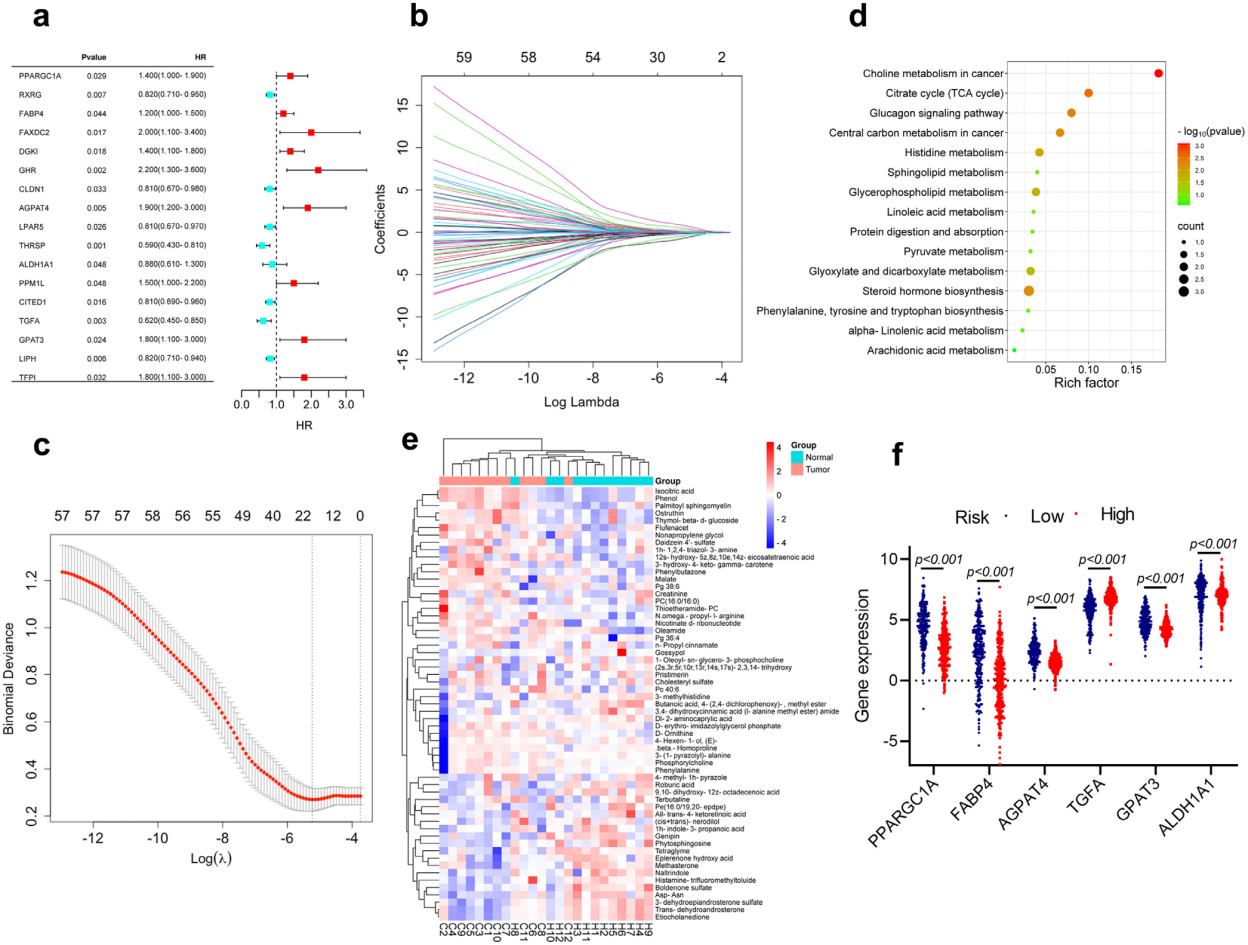
Identifying prognostic risk genes and analyzing their expression based on differentially expressed LMGs by integrating transcriptome and metabolome data. (a) Forest plot of prognostic related LMGs based on univariate Cox regression analysis. (b) LASSO coefficient profiles of the 62 differentially expressed LMGs with non‐zero coefficients were determined by the optimal lambda in the TCGA_THCA dataset. (c) LASSO regression with the screening of optimal parameters (lambda) obtained 16 prognostic genes. (d) KEGG enrichment analysis of the 59 differentially abundant metabolites (DMs). (e) Heatmap illustrating DMs between TC and HC. (f) Differential expression analysis of 6 prognostic signature genes in the high‐ and low‐risk groups.

The four GEO datasets (GSE3678, GSE33630, GSE29265, and GSE3467) include 90 normal and 85 PTC samples, identifying a total of 304 intersecting DEGs, comprising 125 upregulated and 179 downregulated genes (Figure S2a,b). Furthermore, transcriptomic data from TCGA_THCA were obtained, featuring 59 normal and 504 DTC samples. A total of 1603 DEGs were identified, with 895 genes upregulated and 708 genes downregulated (Table S1). Genes associated with lipid metabolism processes were manually screened based on GO and KEGG enrichment analyses (Figure S2c,d), resulting in the selection of 62 differentially expressed LMGs. Among these, 34 were downregulated, and 28 were upregulated (Figure 1a–c). Further enrichment analysis revealed that these genes were linked to the adipocytokine signaling pathway, glycerophospholipid metabolism, PPAR signaling pathway, sphingolipid signaling pathway, as well as fatty acid biosynthesis (Figure 1d). The expression pattern of LMGs in TCGA_THCA was depicted using a heatmap (Figure 1e). Then, the survival analysis was performed using the TCGA_THCA cohort. Univariate Cox regression analysis was employed to explore the potential predictive value of 62 LMGs in TC, identifying 17 LMGs significantly associated with O.S. based on TCGA_THCA (*p* < 0.05; Figure 2a). LASSO regression with tenfold cross‐validation was subsequently executed, resulting in the identification of an optimal lambda value and association with 12 of the 17 prognostic LMGs significantly linked to O.S. (Figure 2b,c).

### 3.2 Identification and Enrichment Analysis of DMs

There was no statistical difference in age, gender, BMI, diabetes, and smoking history between the TC patients and HC. Except for HDL‐C (*p* = 0.010), there was no significant difference in other biochemical indexes between the two groups, including TSH, CEA, Creatinine, ALT, AST, T‐CHOL, TRIG, HDL‐C, LDL‐C, and TRIG/HDL‐C. All TC patients were pathologically diagnosed with PTC post‐operation, with TNM stages I‐II, and 83.33% had the BRAF^V600E^ gene mutation (Table S2). In the metabolomic cohort, both PCA and OPLS‐DA results demonstrated a clear separation between TC and HC groups (Figure S3a–d). In both ESI positive and negative modes, a total of 692 metabolites were identified. Through the adjusted *p* values and *p* value thresholds, 59 metabolites of interest were selected, indicating differences in metabolite abundance between TC and HC. Examples include isocitric acid, malate, phosphorylcholine, phytosphingosine, and trans‐dehydroandrosterone, with characteristics and statistics summarized in Table S5. Importantly, the analysis of major metabolic pathways associated with DMs in TC revealed involvement in pathways such as the citrate cycle (TCA cycle), steroid hormone biosynthesis, glycerophospholipid metabolism, sphingolipid metabolism, and other pathways closely related to lipid metabolism (Figure 2d). Notably, these pathways intersect with those enriched in differentially expressed LMGs. The heatmap analysis of DMs was presented in Figure 2e. Serum TCA cycle intermediates have been investigated in relation to various underlying pathological processes [41, 42]. Isocitric acid and malate as intermediates of the TCA cycle were shown to be more abundant in TC in our analysis. These data suggested a significant involvement of lipid metabolism in the development of TC.

### 3.3 Development and Evaluation of the Prognostic Risk Model

**FIGURE 3:**
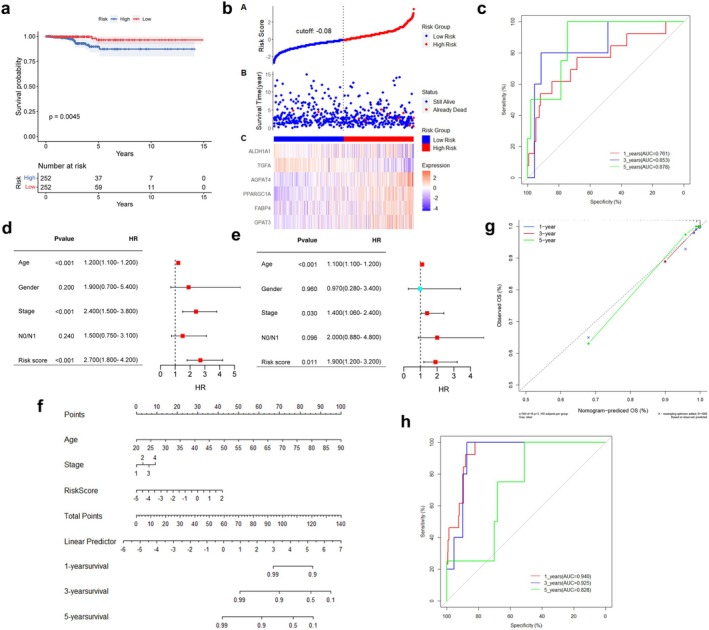
Construction and evaluation of a lipid metabolism‐related prognostic risk model. (a) Kaplan–Meier analysis shows a better overall survival (O.S.) in the low‐risk group than in the high‐risk group in the TCGA_THCA dataset. (b) Survival status, time distribution and the expression levels of 6 LMGs of patients in the high‐ and low‐risk groups. (c) Receiver operating characteristic (ROC) curves showed the performance of the signature in predicting 1‐, 3‐, and 5‐year O.S. (d, e) Univariate Cox regression analyses (d) and multivariate Cox regression analyses (e) regarding clinicopathological characteristics and O.S. of TC patients. (f) Construction of nomogram combining the risk score, age and stage to predict individuals' probability of survival. (g) Calibration curves comparing the nomogram‐predicted 1‐, 3‐, and 5‐year O.S. and actual survival. (h) The ROC curves of the nomogram in 1‐, 3‐, and 5‐year O.S. area under curve (AUC).

To identify key LMGs involved in TC processes, transcriptomes and metabolomes were integrated for analysis. The pathways of 62 LMGs were co‐enriched with the top 20 pathways from the metabolome, resulting in the identification of 29 key LMGs, including 11 overlapping pathways (Figure S3e). Interestingly, 6 genes (FABP4, PPARGC1A, AGPAT4, ALDH1A1, TGFA, and GPAT3) were found to be intersection genes among the 29 key LMGs and the previously identified 12 prognostic genes. Multivariate Cox regression analysis was then performed to investigate the effects of the 6 LMGs, which were subsequently selected as signature genes to establish a prognostic risk model for TC patients (Figure S3f). The prognostic LMGs were represented by the risk score formula: Risk score = (0.414 * GPAT3 + 0.034 * FABP4 + 0.265 * PPARGC1A + 0.451 * AGPAT4–0.266 * TGFA—0.587 * ALDH1A1). THCA patients were stratified into high‐ and low‐risk groups based on the median risk score (−2.067) in the TCGA_THCA cohort, revealing differential expression of the 6 LMGs between the two groups (Figure 2f). K–M analysis revealed that the high‐risk group exhibited lower O.S. compared to the low‐risk group (*p* = 0.0045; Figure 3a). The 1, 3, and 5‐year O.S. rates for the high‐risk group were 93.55%, 93.83%, and 94.42%, respectively, compared to 96.15%, 99.30%, and 98.45% for the low‐risk group (*p* = 0.0144). Figure 3b illustrates the distribution of risk scores and gene expression, correlated with survival status, indicating shorter survival times among patients with high‐risk scores. Moreover, ROC curve analysis was conducted to evaluate the predictive capability of the LMGs prognostic signature. The AUC for the 1‐, 3‐, and 5‐year survival rates were 0.761 (95% CI 0.613–0.909), 0.853 (95% CI 0.687–0.999), and 0.878 (95% CI 0.757–0.999), respectively, suggesting that the 6 LMGs signature exhibited robust predictive ability in TCGA_THCA (Figure 3c).

### 3.4 Prognostic Risk Score Was Associated With Clinical Outcome

Univariable and multivariable Cox regression analyses were employed to examine the relationship between clinical parameters, risk scores, and O.S. The results indicated that the risk score, age, and stage were independent risk factors (*p* < 0.05; Figure 3d,e). Gender and N0/N1 (presence or absence of lymph node metastasis) showed no significance. Subsequently, a nomogram was constructed by integrating these independent prognostic factors to enhance the accuracy and reliability of the predictive model (Figure 3f). The nomogram for predicting 1‐, 3‐, and 5‐year O.S. was calibrated, and the calibration curve revealed excellent concordance between actual observations and nomogram predictions (Figure 3g). The AUC values of the nomogram for predicting 1‐, 3‐, and 5‐year O.S. were 0.940 (95% CI 0.905–0.974), 0.925 (95% CI 0.875–0.976), and 0.828 (95% CI 0.757–0.999), respectively, suggesting that the nomogram exhibited good predictive value for the clinical outcomes of TC patients (Figure 3h).

### 3.5 Association of the Risk Model and Immune Microenvironment

**FIGURE 4:**
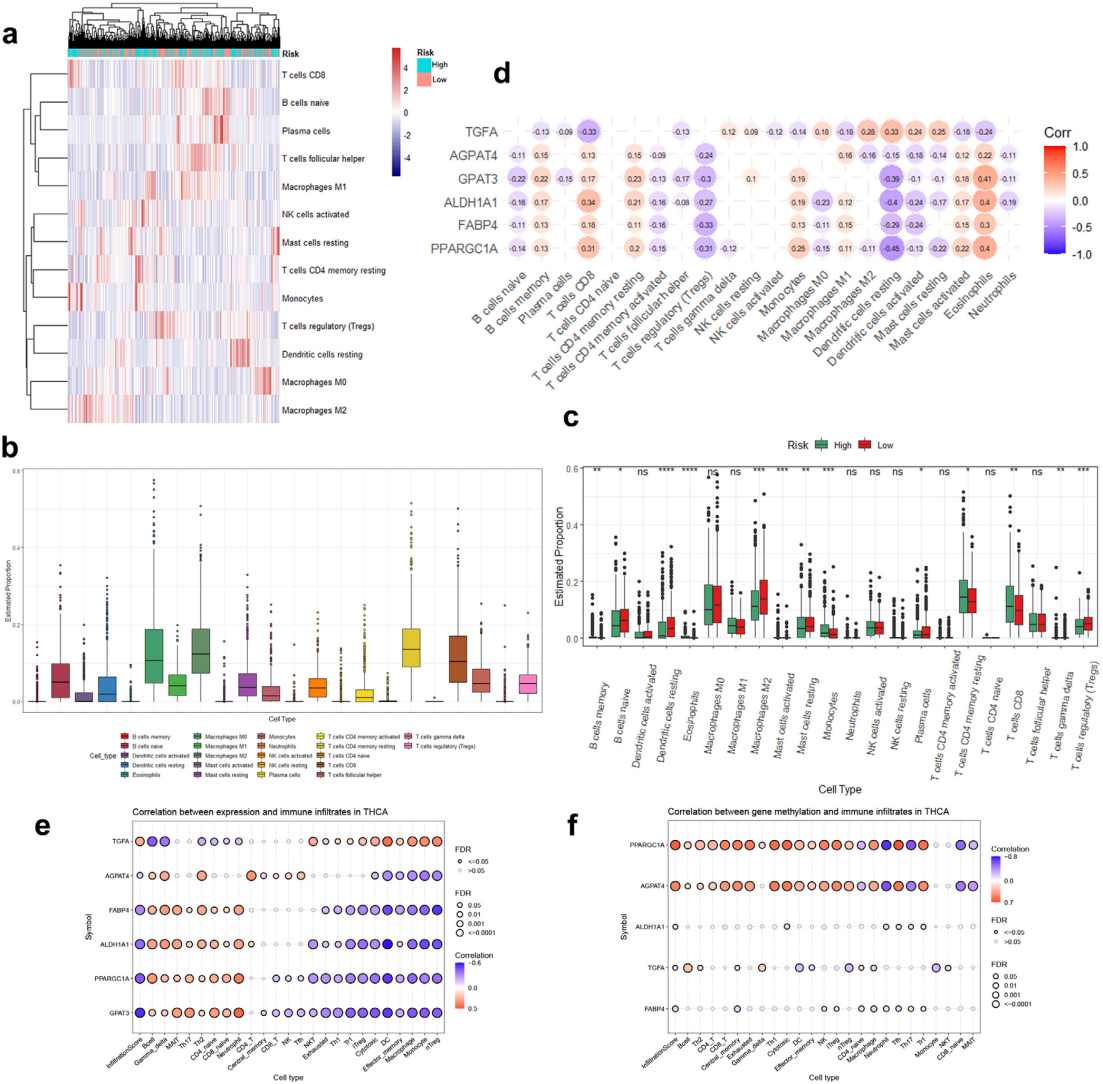
Correlation between the prognostic risk model and tumor‐infiltrating immune cells (TIICs). (a) Comparison of immune cell infiltration (abundance) between high‐ and low‐risk groups. (b) The abundance of various immune cell infiltrates in the TC patients. (c) Differences in distribution of the TIICs between high‐ and low‐risk groups. *p < 0.05, **p < 0.01, ***p < 0.001, ns, not significant. (d) Correlation analysis of the prognostic risk genes and different infiltrating immune cell subtypes. (e) Correlation between 6 LMGs expression and immune infiltration based on the gene set cancer analysis (GSCA) platform. (f) Correlation between 6 LMGs methylation and infiltration levels of immune cells.

Considering the significant impact of lipid metabolic reprogramming on cancer progression and therapeutic response through immune microenvironment remodeling [41], we investigated the relationship between immune‐cell characteristics and the risk model based on LMGs. Using the CIBERSORT algorithm to analyze the abundance of TIICs in the TCGA_THCA cohort, a heatmap was generated to illustrate the differences in immune characteristics (Figure 4a). Figure 4b depicted the abundance of various immune cell infiltrates in TC samples. Further analysis of the differences in the proportion of each of the 22 TIICs between the high‐ and low‐risk groups uncovered significantly higher infiltration levels of B cell memory, monocytes, CD8+ T cells, and CD4+ T cells memory resting in the high‐risk group compared to the low‐risk group (Figure 4c). Notably, the relationship between risk groups and immune cell abundance requires further data validation. Subsequently, we assessed the correlation between the expression of the 6 LMGs and the levels of the 22 TIICs. The results showed a negative correlation between the infiltration of five immune cell types (B cells naïve, CD4+ T cells memory activated, T cells regulatory (Tregs), dendritic cells resting/activated, and macrophages M0) and the expression of five genes (FABP4, PPARGC1A, AGPAT4, ALDH1A1, and GPAT3), while it was positively correlated with B cells memory, CD8+ T cells, CD4+ T cells memory resting, mast cells activated, and eosinophils. The correlation of TGFA was just the opposite (Figure 4d). The results of immunogenomics analysis on the GSCA platform were consistent with our findings, essentially validating our analysis (Figure 4e). Interestingly, the methylation levels of PPARGC1A and AGPAT4 exhibited significant correlations with the infiltration levels of immune cells (Figure 4f).

### 3.6 Association of the Risk Model With Efficacy of Therapeutic Drugs

**FIGURE 5:**
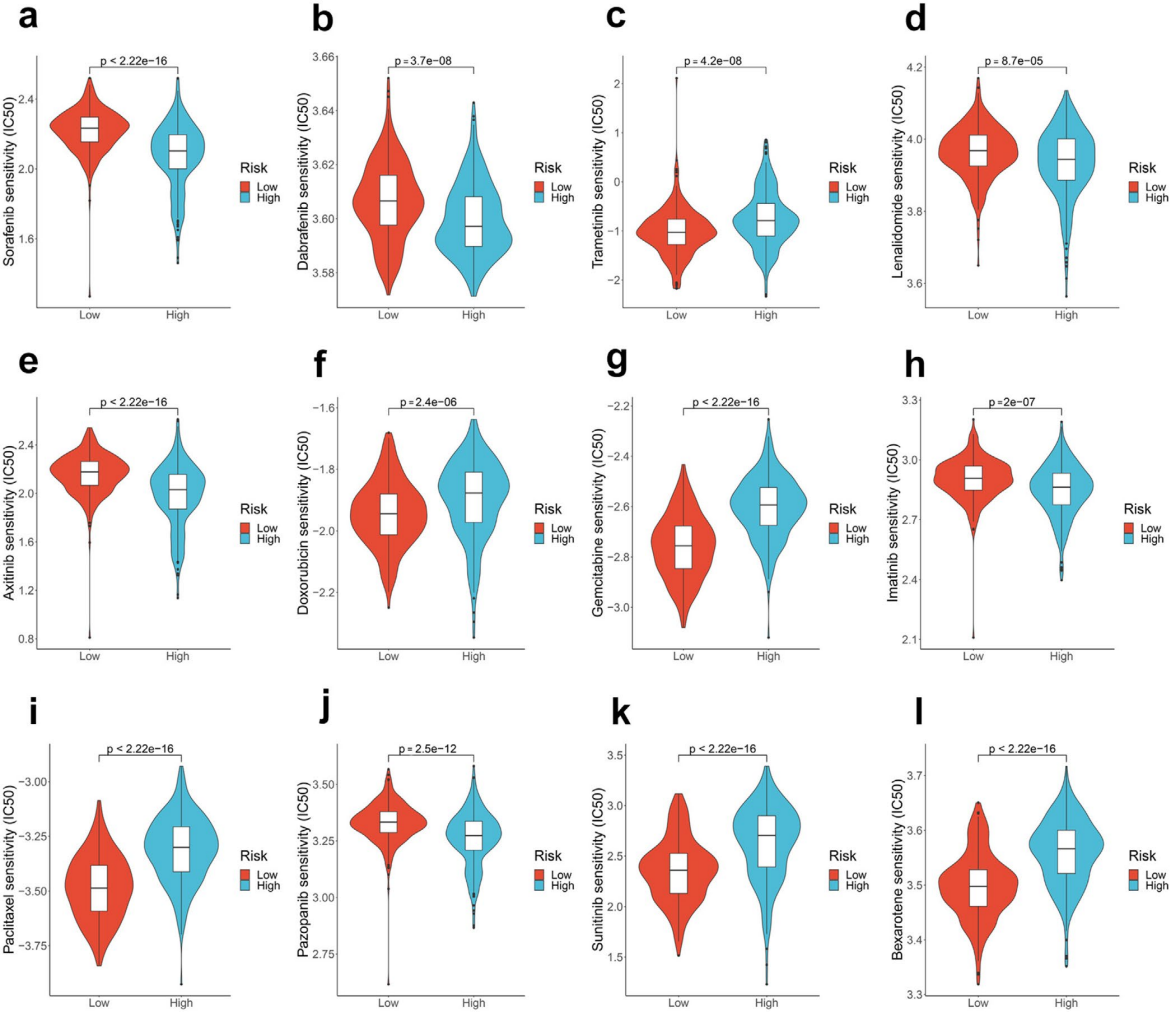
Prediction the sensitivity of potential therapeutic drugs in different risk groups. (a–l) IC_50_ was calculated for sorafenib, dabrafenib, trametinib, lenvatinib, axitinib, doxorubicin, gemcitabine, imatinib, paclitaxel, pazopanib, sunitinib, and bexarotene in the high‐ and low‐risk groups.

We explored the relationship between risk scores and the efficacy of TC treatment‐related drugs by calculating IC_50_. Although some drugs are not FDA approved, they can be considered if clinical trials or other systemic therapies are not available or appropriate [28, 43]. The benefits and risks of available treatment options should be weighed in clinical practice or clinical trials. We observed differences in drug responses between the high‐ and low‐risk groups. The IC_50_ values of sorafenib, dabrafenib, lenvatinib, axitinib, imatinib, and pazopanib were lower in the high‐risk group, indicating that high‐risk patients may benefit more from these drugs (*p* < 0.05; Figure 5a–l). Conversely, trametinib, doxorubicin, gemcitabine, paclitaxel, sunitinib, and bexarotene exhibited higher drug responses in the low‐risk group. These findings imply that patients' responses to potential therapeutic drugs for TC can be predicted based on the risk score.

### 3.7 Potential Mechanisms of the Prognostic Risk Genes in Lipid Metabolism Pathway

**FIGURE 6:**
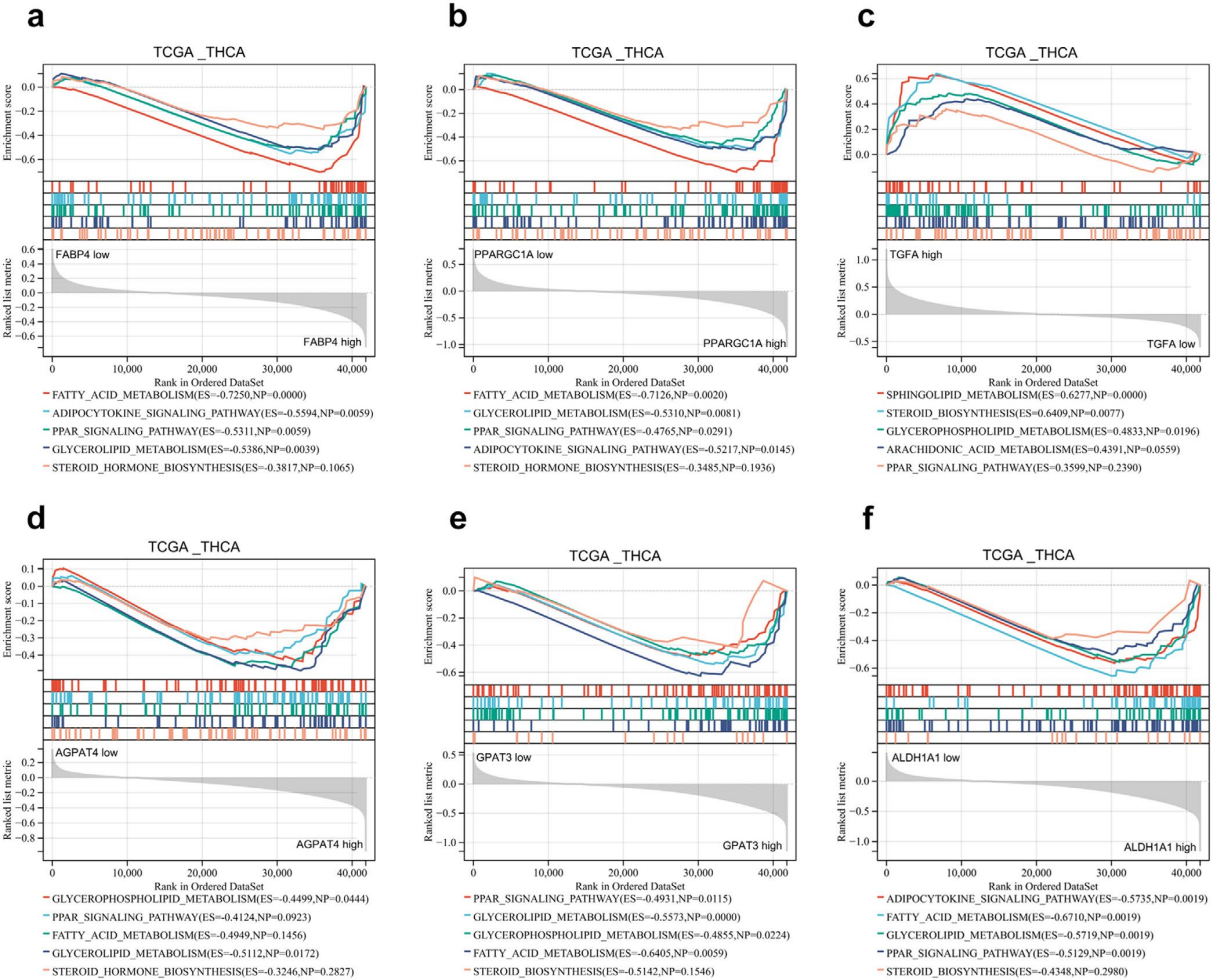
To analyze the potential mechanisms of 6 LMGs affecting the prognosis of TC patients. (a–f) GSEA analysis was performed using TCGA_THCA database to compare the high and low expression of FABP4, PPARGC1A, TGFA, AGPAT4, GPAT3, and ALDH1A1, respectively. ES: Enrichment score. NP: *p* value.

To delve deeper into the potential mechanisms involving the six LMGs, we performed GSEA by comparing the high and low expression levels of these signature genes using the TCGA_THCA database. The analysis revealed significant enrichment of lipid metabolism‐related processes in each prognostic risk gene. Specifically, FABP4, PPARGC1A, AGPAT4, ALDH1A1, and GPAT3 were associated with negative modulation of fatty acid metabolism, the PPAR signaling pathway, steroid biosynthesis, glycerophospholipid metabolism, and the adipocytokine signaling pathway. In contrast, TGFA exhibited the opposite regulatory ability (Figure 6a–f). These results suggested an association between the expression of prognostic risk genes and lipid metabolism signaling pathways in TC patients.

### 3.8 Validation of mRNA and Protein Expressions of Prognostic Signature Genes

**FIGURE 7:**
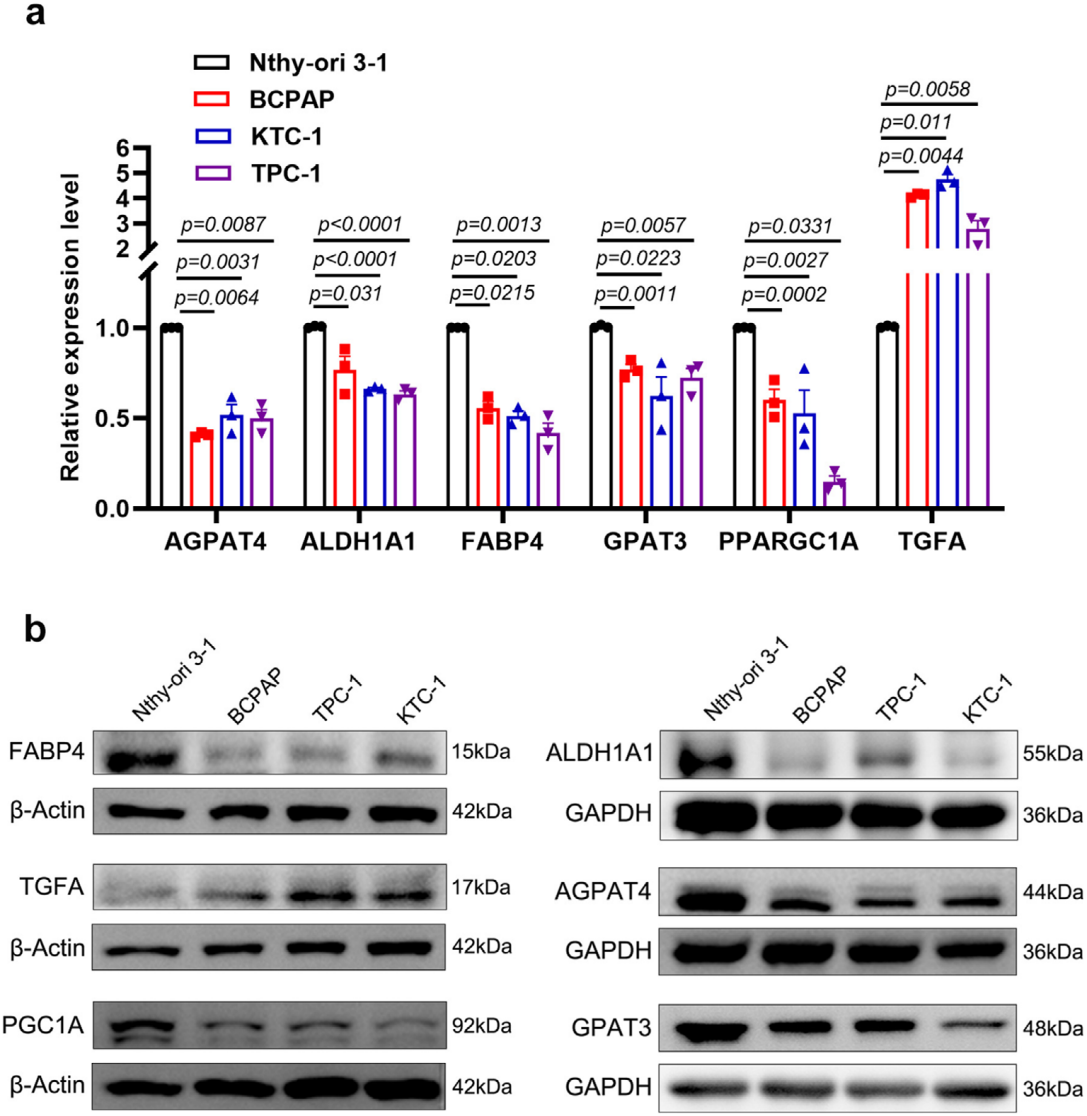
Validation of expression of six prognostic signature genes between TC cells and normal thyroid cell. (a) mRNA relative levels of the 6 LMGs in the thyroid follicular epithelial cell (Nthy‐ori3‐1) and TC cells (BCPAP, KTC‐1, and TPC‐1). Data expressed as the mean ± SEM. *p* values obtained via 2‐tailed unpaired Student's *t* tests. (b) Western blot analysis of 6 LMGs levels in Nthy‐ori3‐1, BCPAP, KTC‐1, and TPC‐1cells, with β‐Actin or GAPDH serving as the loading control.

We conducted RT‐qPCR and Western blot analyses to assess the expression levels of the six LMGs in Nthy‐ori 3–1, BCPAP, KTC‐1, and TPC‐1 cell lines. Compared to the Nthy‐ori 3–1 cell line, TGFA exhibited a marked overexpression at both mRNA and protein levels in BCPAP, KTC‐1, and TPC‐1 cells (*p* < 0.05). Conversely, the expression of FABP4, PPARGC1A (PGC1A), AGPAT4, ALDH1A1, and GPAT3 was significantly lower in BCPAP, KTC‐1, and TPC‐1 cells (Figure 7a,b). These findings validated the results of our bioinformatic analysis, underscoring the reliability of our research.

## 4 Discussion

Previous evidence has indicated that biomarkers related to lipid metabolism are associated with the prognosis of diverse tumor types [42, 44, 45]. However, further exploration is warranted for predictive markers that predict patient prognosis and guide specific targeted therapy. Our study presents a comprehensive approach by integrating metabolomic and transcriptomic analyses derived from TC and HC. Subsequently, through bioinformatic analysis, we developed a prognostic risk model closely associated with O.S., the tumor microenvironment, and potential drug treatment responses. This integrative method could offer valuable insights into the mechanisms underlying TC progression and pave the way for personalized treatment strategies. Recent research has underscored the critical role of lipid metabolism reprogramming in cancer, highlighting a crucial aspect of cancer biology that extends across various types. In liver [46], lung [47], colorectal [48] and ovarian cancers [49], alterations in lipid metabolism have been increasingly acknowledged not merely as a bystander phenomenon but as a pivotal factor in the initiation, progression, and treatment resistance. Wong's research has demonstrated specific mechanisms, notably adenosine deaminases acting on RNA1 (ADAR1), which enhance SCD1 mRNA stability to facilitate lipid droplet formation, leading to worse prognosis and chemoresistance [50]. Additionally, research conducted by Zhao et al. identified a novel regulatory axis involving HKDC1/G3BP1‐PRKDC, which induces gastric cancer metastasis and chemoresistance through reprogramming lipid metabolism [51]. However, the understanding of lipid metabolism‐related genes and their role in TC remains limited. In this study, we conducted a comprehensive evaluation of LMGs and metabolites based on GEO and TCGA_THCA databases, along with an untargeted metabolomics platform. Our integration of lipid metabolism pathways identified six genes—FABP4, PPARGC1A, AGPAT4, ALDH1A1, GPAT3, and TGFA—significantly associated with O.S. in TC patients, leading to the development of a prognostic model. Moreover, considering the influence of clinical variables on prognosis prediction, we employed the risk score as an independent prognostic signature to construct a nomogram incorporating age and stage. Our findings suggested that the risk model, based on multi‐omic analysis, possesses enhanced applicability and practicability, aiding in the identification of high‐risk TC patients.

Our findings highlight alterations in key metabolic pathways, including the tricarboxylic acid (TCA) cycle, glucagon signaling pathway, glycerophospholipid metabolism, steroid hormone biosynthesis, and sphingolipid metabolism. The involvement of these pathways in energy production, macromolecular synthesis, and signal transduction underscores the complexity of metabolic dysregulation in TC. Previous studies have reported associations between the TCA cycle, choline metabolism in cancer, and glycerophospholipid metabolism with tumor growth and invasion across various tumor types [52, 53, 54, 55]. Concurrently, the transcriptomics analysis confirmed alterations in steroid biosynthesis, the glucagon signaling pathway, and glycerophospholipid metabolism in TC tissue. Integration pathway analysis showed significant variations in lipid metabolism between TC and HC, underscoring the importance of lipid‐related pathways in TC progression. Understanding these metabolic disorders holds promise for the development of targeted therapeutic strategies.

Furthermore, our investigation identified six genes crucially involved in lipid‐related biological processes. FABP4, an intracellular lipid chaperone, is downregulated in TC and colorectal cancer [56, 57], correlating with cancer cell proliferation, invasion, and patient prognosis. Notably, FABP4 can act as both a tumor suppressor and an oncogene in different tumors, and is associated with immunotherapy and ferroptosis [58, 59]. PPARGC1A/PGC1A, a pivotal transcriptional coactivator, regulates mitochondrial metabolism. It acts as a tumor suppressor in certain cancers through pathways like WNT/β‐catenin/PDK1, PGC1A/ERRA, and Melatonin/PGC1A/UCP1, regulating metabolism, signaling, and autophagy [60, 61, 62]. AGPAT4 and GPAT3 encode members of the 1‐acylglycerol‐3‐phosphate O‐acyltransferase family, actively participating in de novo phospholipid biosynthesis. Silencing AGPAT4 releases lysophosphatidic acid from cancer cells, polarizing macrophages to an M1‐like phenotype, which promotes CD4+ and CD8+ T cell infiltration and activation, influencing colorectal cancer progression [63]. GPAT3 regulates lipid metabolism, contributing to the inflammatory activation of Kupffer cells [64]. Further investigation is warranted to explore the correlation between the methylation of PGC1A and AGPAT4 and immune infiltration, as, well as their roles in TC prognosis. TGFA encodes a growth factor serving as a ligand for the epidermal growth factor receptor. Researchers have found that high‐fat diets and obesity may differentially modulate TGFA, potentially promoting tumor progression [65]. Elevated levels of ALDH1A1 have been linked to tumor development by influencing the immune system. ALDH1A1's behavior is complex, regulated by various epigenetic processes, and it acts as a tumor suppressor in certain cancers. Our results, consistent with Wei et al., showed ALDH1A1 downregulation in TC patients, associated with tumor progression [66]. Kim et al. suggested ALDH1A1 overexpression reduces proliferation and invasiveness of colorectal cancer cells while promoting metastasis [67]. In this study, FABP4, PPARGC1A, AGPAT4, ALDH1A1, and GPAT3 were identified as downregulated tumor suppressor genes in TC, whereas TGFA was recognized as an oncogene. Tumor cells heavily depend on lipid metabolism to meet their energy demands, facilitate cell growth, generate signaling molecules, and prioritize lipid synthesis to sustain rapid proliferation [16]. Our single‐gene GSEA analysis sheds light on the potential mechanisms through which oncogenes and oncosuppressor genes influence the prognosis of TC patients. For instance, the PPAR signaling pathway, a class of nuclear receptors, is involved in physiological processes like lipid metabolism, cell proliferation, and differentiation [68]. Subsequently, we conducted RT‐qPCR and Western blotting analyses to validate the expression patterns of these genes in TC cells compared to normal thyroid cell. These results lay the groundwork for further in vivo and in vitro functional analyses, facilitating the confirmation of the correlation between candidate genes and lipid metabolism in TC.

Most cancer cells undergo metabolic reprogramming within the immune microenvironment, disrupting the initial conditions and potentially resulting in unfavorable therapeutic outcomes [17]. The accumulation of lipids due to abnormalities in lipid metabolism may contribute to the altered tumor microenvironment, leading to a poor prognosis [69]. Our study indicated a limited infiltration of dendritic cells and macrophages in high‐risk groups, suggesting a deficiency in immune cells that could impede effective anti‐tumor responses. Researchers have proposed that lipid‐laden dendritic cells may struggle to stimulate allogeneic T cells or present tumor‐associated antigens effectively [70, 71]. Afterward, we observed positive or negative correlations between the expression of LMGs and the abundance of immune cells. Qian et al. noted a similar correlation in the immune microenvironment of osteosarcoma patients, suggesting LMGs potential applications for predicting prognosis [72]. Collectively, LMGs can influence TC prognosis by regulating immune cell infiltration. Consequently, a synergistic effect might be anticipated by combining regimens targeting lipid metabolism with strategies to enhance the immune system. Our findings demonstrated that the high‐risk group exhibits lower IC_50_ values for sorafenib, dabrafenib, lenvatinib, axitinib, imatinib, and pazopanib, suggesting that high‐risk patients are more likely to benefit from these drug treatments. Hence, based on the predicted outcomes of these divergent drug treatment responses, potential therapeutic drugs can be selected for TC treatment according to patients' risk scores. It's important to acknowledge that targeted therapy may have limited efficacy due to primary or acquired drug resistance. Targeting aberrant LMGs or lipid metabolites emerges as a promising strategy for anti‐tumor therapy. For instance, reprogramming lipid metabolism through modulating citrate, phosphorylcholine, and linoleic acid has shown promise as a therapeutic strategy for tumors [73, 74, 75]. Importantly, further investigation is warranted to explore the potential synergistic antitumor effect of combining lipid metabolism regulators with small molecular therapeutic drugs in TC.

This study has several limitations. First, the retrospective nature of the transcriptomics cohort, predominantly relying on data from TCGA and GEO databases, emphasizes the importance of validation through prospective studies with larger sample sizes. Second, metabolomics studies are significantly impacted by the coverage of metabolites in databases, potentially leading to the omission of certain metabolites due to low concentrations or absence in the annotation library. Therefore, it is imperative to design and conduct appropriate functional experiments in TC based on the multi‐omics findings, encompassing the lipid metabolism genes, molecules, and signaling pathways identified in this study.

## 5 Conclusions

In summary, this study integrated transcriptomic and metabolomic data to identify key lipid metabolic pathways and LMGs, and developed a prognostic risk model for TC and evaluated potential drug treatments. Our analysis revealed that dysregulated lipid metabolism impedes immune regulation and causes patients to derive varying benefits from therapeutic drugs, impacting prognosis negatively. Consequently, our findings provide novel insights into comprehending the mechanisms of TC lipid metabolism disorder and advocate for individualized therapy to enhance patient prognosis.

## Author Contributions

**Yuqin Tu:** conceptualization (lead), data curation (equal), investigation (equal), methodology (lead), software (equal), visualization (lead), writing – original draft (lead). **Yanchen Chen:** investigation (equal), methodology (equal), software (equal), validation (equal), visualization (equal). **Linlong Mo:** data curation (equal), methodology (equal), software (equal). **Guiling Yan:** investigation (equal), methodology (equal), software (equal). **Jingling Xie:** investigation (equal), methodology (equal), software (equal). **Xinyao Ji:** methodology (equal), software (equal). **Shu Chen:** methodology (equal), software (equal). **Changchun Niu:** conceptualization (equal), formal analysis (equal), methodology (equal), software (equal). **Pu Liao:** conceptualization (equal), data curation (equal), investigation (equal), methodology (equal), project administration (lead), supervision (lead), writing – original draft (equal), writing – review and editing (equal).

## Ethics Statement

The collection and use of human serum samples were approved by the Medical Ethics Committee of Chongqing General Hospital (Approval No. KYS2022‐029‐01). We applied for and received a waiver of informed consent. Patient anonymity and confidentiality were strictly maintained throughout the study.

## Conflicts of Interest

The authors declare no conflicts of interest.

## Supporting information


Data S1.

## Data Availability

The data utilized to support the metabolomics results of this study are restricted by the Clinical Research Ethics Committee of Chongqing General Hospital to protect patient privacy. Other data supporting the findings of this study are openly available in public databases. The data can be accessed through the Gene Expression Omnibus database (https://www.ncbi.nlm.nih.gov/geo/) and The Cancer Genome Atlas (https://portal.gdc.cancer.gov/).
