# Supplementary material for: Multi‐Omic Analysis Reveals a Lipid Metabolism Gene Signature and Predicts Prognosis and Chemotherapy Response in Thyroid Carcinoma
Source: Cancer Med. 2025 Mar 22;14(6):e70819. doi: 10.1002/cam4.70819 (PMC11928767; doi:10.1002/cam4.70819)
Supplement: Supplementary file 1 — Data S1. [file CAM4-14-e70819-s001.docx]

**Multi-Omic Analysis Reveals a Lipid Metabolism Gene Signature and Predicts Prognosis and Chemotherapy Response in Thyroid Carcinoma**

Yuqin Tu^1,2^, Yanchen Chen^3^, Linlong Mo^4^, Guiling Yan^1,2^, Jingling Xie^1,2^, Xinyao Ji^1,2^, Shu Chen^1,2^, Changchun Niu^1,2^, Pu Liao^1,2*^

**Table S1.** **Analysis of the GEO and TCGA databases of thyroid carcinoma.**

| GEO datasets | Tumor | Normal | Total | Platforms | Total DEGs | Up | Down |
| --- | --- | --- | --- | --- | --- | --- | --- |
| GSE3678 | 7 | 7 | 14 | GPL570 | 594 | 252 | 342 |
| GSE33630 | 49 | 45 | 94 | GPL570 | 1160 | 635 | 525 |
| GSE29265 | 20 | 29 | 49 | GPL570 | 914 | 414 | 500 |
| GSE3467 | 9 | 9 | 18 | GPL570 | 760 | 369 | 391 |
| TCGA_THCA | 504 | 59 | 563 | RNA-Seq | 1603 | 895 | 708 |

**Table S2.** **Clinical characteristics of subjects.**

| Characteristics | | TC | | HC | | *p* value | |
| --- | --- | --- | --- | --- | --- | --- | --- |
| Subjects | | 12 | | 12 | | - | |
| Age (years) | | 41.58 ± 8.25 | | 39.5 ± 11.84 | | 0.773 | |
| Gender ratio | | 3/9 | | 4/8 | | 0.660 | |
| Male | | 3 (25%) | | 4 (33.33%) | |  | |
| Female | | 9 (75%) | | 8 (66.67%) | |  | |
| BMI | | 23.89 ± 3.28 | | 23.04 ± 5.14 | | 0.419 | |
| Diabetes | | 0/12 | | 0/12 | | 1.000 | |
| Yes | | 0 (0%) | | 0 (0%) | |  | |
| No | | 12 (100%) | | 12 (100%) | |  | |
| Smoking | | 1/12 | | 1/12 | | 1.000 | |
| Yes | | 1 (8.33%) | | 1 (8.33%) | |  | |
| No | | 11 (91.67%) | | 11 (91.67%) | |  | |
| TSH | | 2.46 ± 1.66 | | 2.56 ± 0.76 | | 0.419 | |
| CEA | | 1.10 ± 0.46 | | 1.41 ± 0.81 | | 0.488 | |
| Creatinine | | 63.64 ± 10.05 | | 64.53 ± 14.40 | | 1.000 | |
| ALT | | 20.50 ± 11.93 | | 20.98 ± 9.78 | | 0.488 | |
| AST | | 19.58 ± 5.72 | | 22.53 ± 8.24 | | 0.402 | |
| T-CHOL | | 4.79 ± 0.90 | | 5.15 ± 0.59 | | 0.386 | |
| TRIG | | 1.51 ± 0.60 | | 1.31 ± 0.36 | | 0.623 | |
| HDL-C | | 1.21 ± 0.24 | | 1.46 ± 0.24 | | 0.010^*^ | |
| LDL-C | | 2.38 ± 0.54 | | 2.64 ± 0.50 | | 0.194 | |
| TRIG/ HDL-C | | 1.28 ± 0.53 | | 0.91 ± 0.27 | | 0.094 | |
| Histological type | |  | |  | |  | |
| PTC | | 12 (100%) | | - | | - | |
| BRAF^V600E^ mutation | |  | |  | |  | |
| Yes | | 10 (83.33%) | | - | | - | |
| No | | 2 (16.67%) | | - | | - | |
| Status of the TC patients | |  | |  | |  | |
| at diagnosis | | 0 (0%) | | - | | - | |
| following surgery | | 12 (100%) | | - | | - | |
| Tumor diameter (cm) | |  | |  | |  | |
| ≤1 cm | | 8 (66.67%) | | - | | - | |
| >1 cm | | 4 (33.33%) | | - | | - | |
| Tumor stage (TNM) | |  | |  | |  | |
| I, II | | 12 (100%) | | - | | - | |
| III, IV | | 0 (0%) | | - | | - | |
| Lymph node metastasis | |  | |  | |  | |
| Yes | | 7 (58.33%) | | - | | - | |
| No | | 5 (41.67%) | | - | | - | |

TC, thyroid carcinoma; HC, healthy control; BMI, Body Mass Index; TSH, thyroid stimulating hormone; CEA, carcinoembryonic antigen; ALT, alanine transaminase; AST, aspartate transaminase; T-CHOL, total cholesterol; TRIG, triglyceride; HDL-C, high density lipoprotein cholesterol; LDL-C, low-density lipoprotein cholesterol; TRIG/HDL-C, the ratio of triglyceride to high density lipoprotein cholesterol; PTC, papillary thyroid carcinoma; TNM, tumor node metastasis classification; -, Not applicable; *, *p* < 0.05.

**Table S3.** **Primers for Real-Time quantitative PCR.**

| Genes | Forward primer | Reverse primer |
| --- | --- | --- |
| FABP4 | ACTGGGCCAGGAATTTGACG | CTCGTGGAAGTGACGCCTT |
| PPARGC1A | TCTGAGTCTGTATGGAGTGACAT | CCAAGTCGTTCACATCTAGTTCA |
| TGFA | AGGTCCGAAAACACTGTGAGT | AGCAAGCGGTTCTTCCCTTC |
| AGPAT4 | CTCAGGGCTAATCATCAACACC | GCTTGAGATGCAATAGGACAGT |
| GPAT3 | CGCTGGTTCTCGGCTTCAT | TGGCCCACTCTAAAGTTTTCAC |
| ALDH1A1 | GGGGCAGCCATTTCTTCTCA | CATTGTCCAAGTCGGCATCAG |

**Table S4.** **The antibody information.**

| Name | Supplier | Cat no. |
| --- | --- | --- |
| FABP4 | HUABIO, China | ET1703-98 |
| PPARGC1A | Abcam, USA | ab191838 |
| TGFA | HUABIO, China | ET7107-40 |
| AGPAT4 | HUABIO, China | ER63869 |
| GPAT3 | HUABIO, China | ER63871 |
| ALDH1A1 | Wanleibio, China | WL02762 |
| β-Actin | HUABIO, China | M1210-2 |
| GAPDH | Proteintech, China | 10494-1-AP |
| Goat Anti-Mouse IgG H&L | HUABIO, China | HA1006 |
| Goat Anti- Rabbit IgG H&L | HUABIO, China | HA1001 |

**Table S5.** **59 differentially abundant metabolites.**

| Name | ID | M/Z | RT(s) | Adduct | *p* | adj.*p*.Val |
| --- | --- | --- | --- | --- | --- | --- |
| (2s,3r,5r,10r,13r,14s,17s)-2,3,14-trihydroxy-10,13-dimethyl-17-[(2r,3r,5r)-2,3,6-trihydroxy-5,6-dimethylheptan-2-yl]-2,3,4,5,9,11,12,15,16,17-decahydro-1h-cyclopenta[a]phenanthren-6-one | M493T36 | 493.33486 | 36.2421 | [M-H]- | 0.016 | 0.512 |
| Nicotinate d-ribonucleotide | M193T39 | 192.98043 | 38.9583 | [M-H-C6H7NO3]- | 0.011 | 0.512 |
| Palmitoyl sphingomyelin | M704T916 | 703.57474 | 916.0755 | [M+H]+ | 0.024 | 0.512 |
| Isocitric acid | M173T33_3 | 172.99059 | 32.625 | [M-H-H2O]- | 0.007 | 0.512 |
| Phenol | M93T33 | 93.03302 | 32.6368 | [M-H]- | 0.007 | 0.512 |
| 4-methyl-1h-pyrazole | M83T150 | 83.06103 | 150.007 | [M+H]+ | 0.021 | 0.512 |
| 9,10-dihydroxy-12z-octadecenoic acid | M297T184 | 297.24219 | 184.4665 | [M+H-H2O]+ | 0.038 | 0.530 |
| Roburic acid | M439T76 | 439.3786 | 76.43445 | [M-H]- | 0.024 | 0.512 |
| Nonapropylene glycol | M541T61 | 541.39426 | 60.9109 | [M+H]+ | 0.022 | 0.512 |
| Flufenacet | M152T919 | 152.04729 | 918.54 | [M+H-C6H7F3N2OS]+ | 0.048 | 0.557 |
| Thioetheramide-PC | M759T211 | 758.56902 | 210.678 | (M+Na)+ | 0.044 | 0.531 |
| (cis+trans)-nerodilol | M109T805 | 109.10146 | 804.9315 | [M+H-C7H14O]+ | 0.033 | 0.512 |
| Malate | M133T387 | 133.01318 | 387.4 | [M-H]- | 0.020 | 0.512 |
| 3-methylhistidine | M170T410 | 170.09279 | 410.479 | [M+H]+ | 0.033 | 0.512 |
| Eplerenone hydroxy acid | M431T85 | 431.21026 | 84.5259 | [M-H]- | 0.026 | 0.512 |
| Tetraglyme | M223T76 | 223.15397 | 76.02415 | [M+H]+ | 0.009 | 0.512 |
| Histamine-trifluoromethyltoluide | M381T52 | 381.17393 | 52.4652 | [M-H]- | 0.034 | 0.512 |
| Butanoic acid, 4-(2,4-dichlorophenoxy)-, methyl ester | M101T192 | 101.07138 | 191.884 | [M+H-C6H4Cl2O]+ | 0.012 | 0.512 |
| Pc 40:6 | M893T230 | 892.60755 | 230.221 | [M+CH3COOH-H]- | 0.039 | 0.530 |
| Dl-2-aminocaprylic acid | M160T306_1 | 160.13314 | 305.851 | [M+H]+ | 0.007 | 0.512 |
| 3,4-dihydroxycinnamic acid (l-alanine methyl ester) amide | M232T47_2 | 232.07686 | 47.2558 | [M-H-CH4O]- | 0.037 | 0.530 |
| Phytosphingosine | M318T172 | 318.30008 | 171.536 | [M+H]+ | 0.017 | 0.512 |
| 1h-indole-3-propanoic acid | M188T163 | 188.07092 | 162.59 | [M-H]- | 0.022 | 0.512 |
| All-trans-4-ketoretinoic acid | M269T77 | 269.21186 | 76.7825 | [M-H-CO2]- | 0.045 | 0.531 |
| N.omega.-propyl-l-arginine | M102T558 | 102.09173 | 557.834 | [M+H-C5H9O2N]+ | 0.021 | 0.512 |
| Gossypol | M501T791 | 501.19862 | 791.037 | [M+H-H2O]+ | 0.025 | 0.512 |
| Creatinine | M114T284 | 114.06641 | 283.612 | [M+H]+ | 0.037 | 0.530 |
| Terbutaline | M208T286 | 208.13313 | 285.5425 | [M+H-H2O]+ | 0.028 | 0.512 |
| 3-dehydroepiandrosterone sulfate | M367T39_1 | 367.15817 | 38.6426 | [M-H]- | 0.013 | 0.512 |
| Etiocholanedione | M253T39 | 253.19499 | 38.75905 | [M+H-2H2O]+ | 0.010 | 0.512 |
| Trans-dehydroandrosterone | M271T39 | 271.20551 | 38.8624 | [M+H-H2O]+ | 0.011 | 0.512 |
| Asp-Asn | M246T55 | 246.0745 | 54.5683 | [M-H]- | 0.029 | 0.512 |
| Boldenone sulfate | M365T40 | 365.14264 | 40.0118 | [M-H]- | 0.027 | 0.512 |
| Naltrindole | M413T53 | 413.2 | 52.91845 | [M-H]- | 0.031 | 0.512 |
| Methasterone | M301T42 | 301.25243 | 42.2556 | [M+H-H2O]+ | 0.029 | 0.512 |
| Pe(16:0/19,20-epdpe) | M779T59 | 778.51341 | 58.6784 | [M-H]- | 0.024 | 0.512 |
| Genipin | M249T903 | 249.07643 | 903.45 | [M+Na]+ | 0.011 | 0.512 |
| PC(16:0/16:0) | M795T197 | 794.60504 | 197.492 | (M+CH3COO+2H)+ | 0.033 | 0.512 |
| Pg 38:6 | M793T77 | 793.49698 | 76.8914 | [M-H]- | 0.015 | 0.512 |
| n-Propyl cinnamate | M208T223 | 208.13303 | 222.55 | (M+NH4)+ | 0.003 | 0.512 |
| Ostruthin | M297T41 | 297.15244 | 40.9868 | [M-H]- | 0.025 | 0.512 |
| Thymol-beta-d-glucoside | M311T41 | 311.1682 | 41.04225 | [M-H]- | 0.043 | 0.531 |
| 1h-1,2,4-triazol-3-amine | M85T289 | 85.02893 | 289.092 | [M+H]+ | 0.033 | 0.512 |
| 3-(1-pyrazolyl)-alanine | M156T333 | 156.07678 | 333.3555 | [M+H]+ | 0.028 | 0.512 |
| Pg 36:4 | M770T59 | 769.50257 | 59.1112 | [M-H]- | 0.043 | 0.531 |
| .beta.-Homoproline | M130T469 | 130.0866 | 468.9465 | (M+H)+ | 0.042 | 0.531 |
| 4-Hexen-1-ol, (E)- | M83T230 | 83.08606 | 230.4385 | (M+H-H2O)+ | 0.041 | 0.531 |
| D-Ornithine | M133T433 | 133.09728 | 433.009 | (M+H)+ | 0.039 | 0.530 |
| Phenylalanine | M120T241 | 120.08089 | 240.674 | [M+H-CH2O2]+ | 0.004 | 0.512 |
| D-erythro-imidazolylglycerol phosphate | M111T195 | 111.0556 | 194.5975 | [M+H-CH5O5P]+ | 0.008 | 0.512 |
| Phosphorylcholine | M184T304 | 184.07339 | 303.971 | (M+H)+ | 0.034 | 0.512 |
| 1-Oleoyl-sn-glycero-3-phosphocholine | M544T213 | 544.33931 | 213.214 | (M+Na)+ | 0.026 | 0.512 |
| Daidzein 4'-sulfate | M333T42 | 333.00687 | 42.1283 | [M-H]- | 0.016 | 0.512 |
| Phenylbutazone | M309T40 | 309.15678 | 40.2414 | [M+H]+ | 0.014 | 0.512 |
| 3-hydroxy-4-keto-gamma-carotene | M567T55_2 | 567.41848 | 54.9192 | [M+H]+ | 0.006 | 0.512 |
| Cholesteryl sulfate | M465T36 | 465.30371 | 35.52405 | [M-H]- | 0.010 | 0.512 |
| Pristimerin | M463T35 | 463.28809 | 35.4945 | [M-H]- | 0.001 | 0.502 |
| 12s-hydroxy-5z,8z,10e,14z-eicosatetraenoic acid | M319T60 | 319.22736 | 59.8198 | [M-H]- | 0.044 | 0.531 |
| Oleamide | M282T57 | 282.27895 | 57.18365 | [M+H]+ | 0.028 | 0.512 |

**Figure S1. Flow-chart of datasets analysis.**


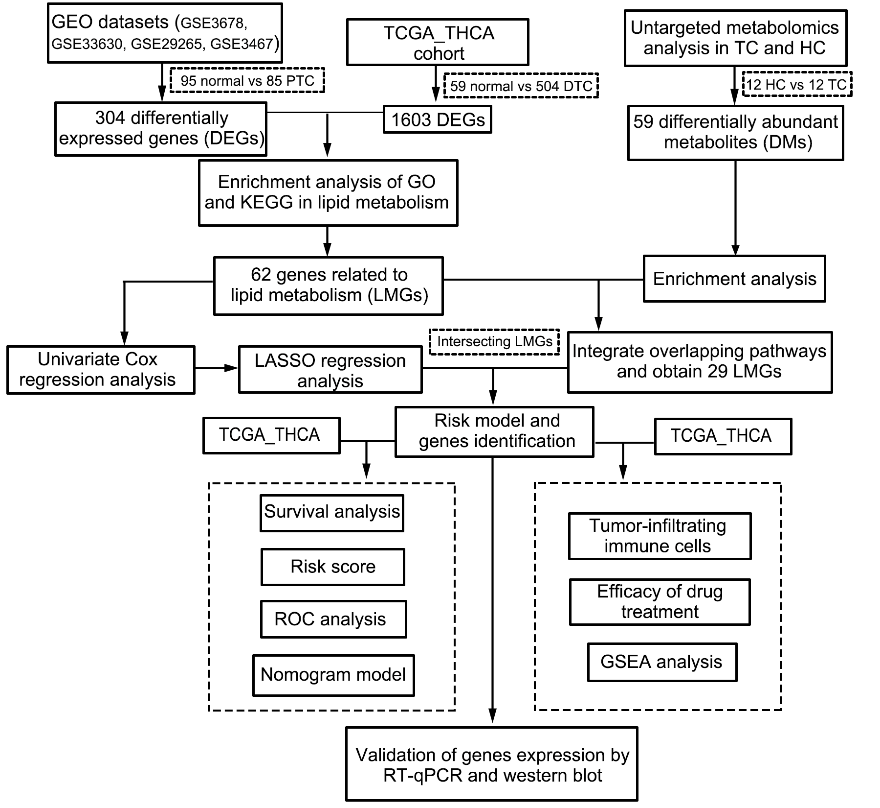


**Figure S2.** **Differential expression analysis and functional annotation in GEO and TCGA_THCA databases.**


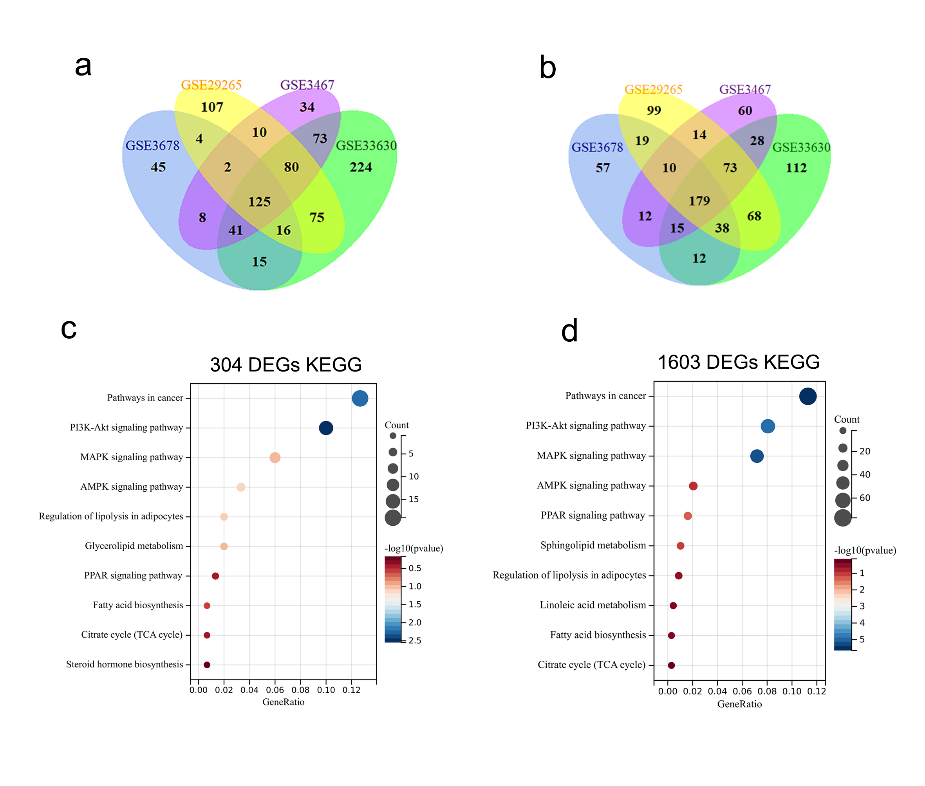


(a-b) Screening of DEGs in datasets GSE3678, GSE33630, GSE29265, and GSE3467. The overlapping regions indicate the commonly upregulated DEGs (a). The overlapping regions indicate the commonly downregulated DEGs (b). (c) KEGG pathway analysis of overlapping DEGs in four GEO datasets. (d) KEGG pathway analysis of DEGs in TCGA_THCA.

**Figure S3.** **Metabolomics data analysis, with transcriptome and metabolome data integration analysis.**


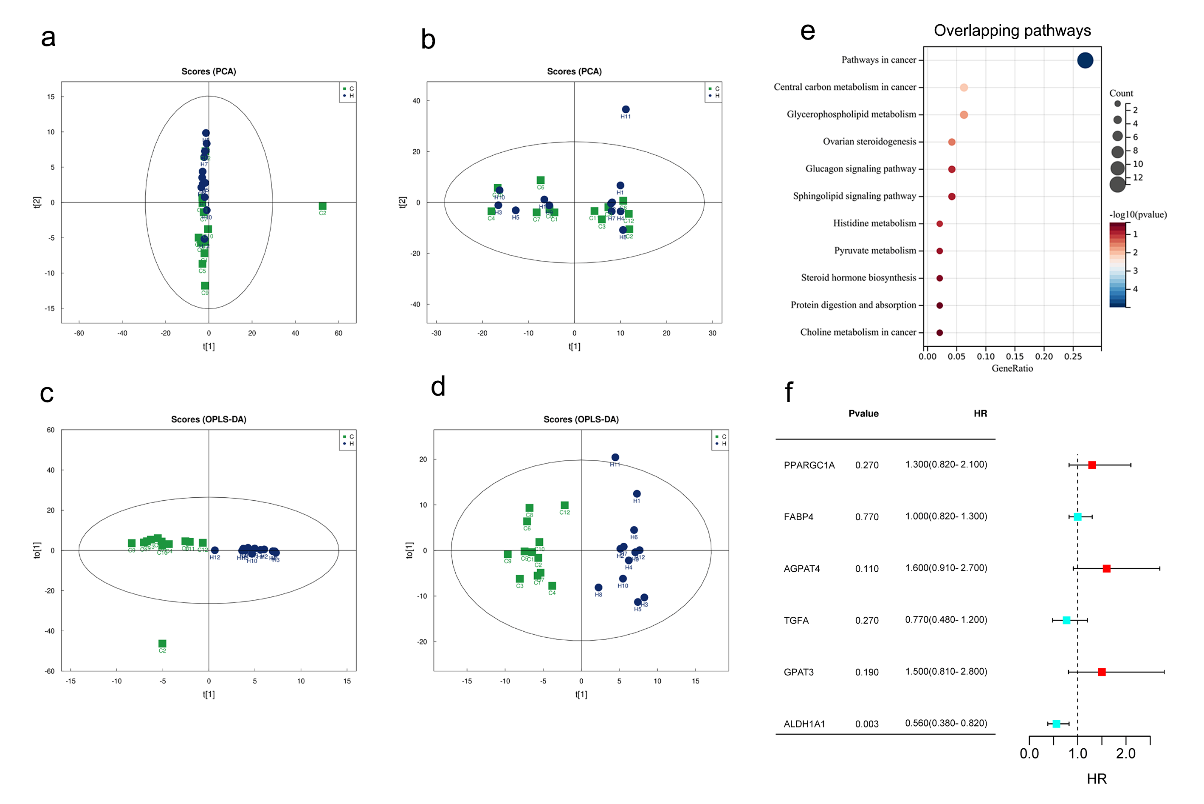


(a-d) Score scatter plots of principal component analysis (PCA); C1-C12: thyroid cancer patient; H1-H12: healthy control. (a-b) and orthogonal partial least-squares discriminant analysis (OPLS-DA) (a-b) between TC and HC. (e) Overlapping lipid metabolism pathways were identified after co-enrichment analysis between 62 LMGs and 59 DMs. (f) Multivariate COX regression analysis of 6 LMGs.
